# Contribution of non-steroidal anti-inflammatory drugs to breast cancer treatment: *In vitro* and *in vivo* studies

**DOI:** 10.14202/vetworld.2024.1052-1072

**Published:** 2024-05-15

**Authors:** Tiago Ferreira, Ana I. Faustino-Rocha, Vítor M. Gaspar, Rui Medeiros, João F. Mano, Paula A. Oliveira

**Affiliations:** 1 Centre for the Research and Technology of Agro-Environmental and Biological Sciences (CITAB), University of Trás-os-Montes and Alto Douro, 5000-801 Vila Real, Portugal; 2 Institute for Innovation, Capacity Building and Sustainability of Agri-Food Production (Inov4Agro), UTAD, 5000–801 Vila Real, Portugal; 3 Molecular Oncology and Viral Pathology Group, Research Center of IPO Porto (CI-IPOP)/RISE@CI-IPOP (Health Research Network), Portuguese Oncology Institute of Porto (IPO Porto), Porto Comprehensive Cancer Center (Porto. CCC), 4200–072 Porto, Portugal; 4 Department of Chemistry, Aveiro Institute of Materials (CICECO), University of Aveiro, Campus Universitário de Santiago, 3810–193, Aveiro, Portugal; 5 Department of Zootechnics, School of Sciences and Technology, University of Évora, Évora 7004-516, Portugal; 6 Comprehensive Health Research Center, 7004–516 Évora, Portugal; 7 Faculty of Medicine of the University of Porto, 4200–319 Porto, Portugal; 8 Department of Research, Portuguese League against Cancer-Regional Nucleus of the North (Liga Portuguesa Contra o Cancro-Núcleo Regional do Norte), 4200–177 Porto, Portugal; 9 Virology Service, IPO Porto, 4200-072 Porto, Portugal; 10 Biomedical Research Center (CEBIMED), Faculty of Health Sciences, Fernando Pessoa University, Porto 4249-004, Portugal

**Keywords:** breast cancer, chemoprevention, COX-2, cyclooxygenase, experimental studies, NSAIDs

## Abstract

Chronic inflammation plays a crucial role in carcinogenesis. High levels of serum prostaglandin E2 and tissue overexpression of cyclooxygenase-2 (COX-2) have been described in breast, urinary, colorectal, prostate, and lung cancers as being involved in tumor initiation, promotion, progression, angiogenesis, and immunosuppression. Non-steroidal anti-inflammatory drugs (NSAIDs) are prescribed for several medical conditions to not only decrease pain and fever but also reduce inflammation by inhibiting COX and its product synthesis. To date, significant efforts have been made to better understand and clarify the interplay between cancer development, inflammation, and NSAIDs with a view toward addressing their potential for cancer management. This review provides readers with an overview of the potential use of NSAIDs and selective COX-2 inhibitors for breast cancer treatment, highlighting pre-clinical in vitro and in vivo studies employed to evaluate the efficacy of NSAIDs and their use in combination with other antineoplastic drugs.

## Introduction

Inflammation has been closely associated with cancer since the 19^th^ century and plays a critical role in tumor initiation, promotion, and progression [1–3]. Cyclooxygenase-1 (COX-1) was identified as a therapeutic target of non-steroidal anti-inflammatory drugs (NSAIDs) in 1971 by British pharmacologist Vane (1927-2004). Using a guinea pig lung homogenate, acetylsalicylic acid decreased the production of prostaglandins (PGs), which are inflammatory mediators, through COX inhibition [4]. Vane received the Nobel Prize in Physiology or Medicine in 1982 for his work on PGs and related substances [5]. In 1991, Xie *et al*. discovered COX-2 [6], which is also responsible for the synthesis of inflammatory mediators [7]. COX-2 is involved not only in inflammation but also in cancer development and is overexpressed in many tumors, particularly breast cancer [8–10]. Chemoprevention refers to the administration of chemical, natural, or synthetic substances that reverse, suppress, or prevent tumor progression [11]. COX inhibitors, particularly NSAIDs targeting COX-2, have been shown to be potential chemopreventive and therapeutic approaches in malignant neoplasms [12, 13].

Epidemiological, experimental, and clinical studies suggest that the administration of NSAIDs reduces the risk of developing cancer due to their ability to reduce the synthesis of inflammatory mediators. NSAIDs have antiproliferative, proapoptotic, antimetastatic, angiogenesis-inhibiting, and immunomodulatory effects because inflammatory mediators are among the hallmarks of cancer [14–16].

This review aims to provide an overview of the potential use of NSAIDs and selective COX-2 inhibitors in the treatment of breast cancer, namely pre-clinical *in vitro* and *in vivo* studies used in the treatment of breast cancer. This study will also be useful for researchers working in this field in terms of experimental design, understanding the results obtained, and providing valuable tools for future research.

## Role of Inflammation in Carcinogenesis

Inflammation is a complex biological response by homeostasis to cellular and tissue damage. It is stimulated by microbial pathogen infections (viruses, bacteria, or parasites) [17, 18], physical injuries [19], and exposure to chemical compounds [14]. Inflammation can be classified as acute, subacute, or chronic [20]. Acute inflammation is the initial response to injury and results from innate immunity. If acute inflammation does not occur, subacute inflammation develops and leads to chronic inflammation. Subacute inflammation usually persists for several weeks. Chronic inflammation will be established if inflammation persists for a long time [21]. Chronic inflammation is characterized by continuous secretion of growth factors and cytokines by mononuclear cells (e.g., lymphocytes and macrophages) [2, 22]. The upregulation of proinflammatory molecules can lead to DNA damage, creating a microenvironment that supports cell proliferation and predisposes the subject to cancer [23]. Inflammatory cells and their inflammatory mediators, such as cytokines (i.e., tumor-necrosis factor-α [TNF-α], interleukin-6 [IL-6], transforming growth factor-β, and IL-10), chemokines (CC chemokine receptors), lipid mediators (PGs and leukotrienes), inducible nitric oxide synthase, nuclear factor kappa-light-chain-enhancer of activated B-cells, hypoxia-inducible factor 1-alpha, and signal transducer and activator of transcription 3, are the main molecules simultaneously involved in inflammation and cancer [24, 25]. In 1863, Rudolf Virchow (1821–1902) first hypothesized a possible relationship between chronic inflammation and cancer [26]. In 1968, Harold Dvorak observed through histological evidence that inflammation and cancer share common features such as proliferation, cell survival, induced angiogenesis, and migration [27]. Approximately 20% of human cancers are related to chronic inflammation. Therefore, NSAIDs against chronic inflammation may be a relevant therapeutic strategy against breast cancer [1]. Inflammation is associated with carcinogenesis through intrinsic and extrinsic pathways. The intrinsic pathway is mediated by genetic alterations, including oncogene activation, chromosomal rearrangement or amplification, and tumor suppressor gene inactivation, while the extrinsic pathway is activated by inflammatory stimuli [14]. Therefore, the inflammatory state contributes to tumor development through different mechanisms such as induction of genomic instability, changes in epigenetic agents, stimulation of cellular proliferation, and resistance to apoptosis, angiogenesis, invasion, and metastasis [24].

## COXs and Cancer

PG-endoperoxide synthase or PG H synthase, colloquially referred to as COX, is a dimer of 70–72 kDa subunits identified by Vane in 1971. Twenty years later, a second isoform (COX-2), which differs from the first isoform and encodes a different gene, was discovered. These isoenzymes have been renamed COX-1 and COX-2 [4, 6]. To date, three isoenzymes have been identified: COX-1, COX-2, and COX-3 [28]. COX-1 and COX-2 are heme peroxidase enzymes responsible for the bioconversion of arachidonic acid to various eicosanoids such as prostanoids, lipoxins, leukotrienes, and resolving [7]. Each COX monomer comprises a short N-terminal epidermal growth factor, a membrane-binding domain, and a globular C-terminal catalytic domain, where peroxidase and COX active sites are present [29, 30]. Homodimers are membrane-bound enzymes located in the bilayer of the endoplasmic reticulum and nuclear envelope [31].

COX-1 and COX-2 have similar structures and lengths with 576 and 581 amino acids, respectively [32], exhibiting approximately 60% homology in their amino acid sequence within the same species. However, there are some differences between these reports, namely, that COX-1 contains an 8-amino acid insert in the N-terminal region of the enzyme that is not found in COX-2, whereas COX-2 has 18 amino acids at the C-terminal end that are not found in COX-1 [33]. In addition, there are minor differences in catalytic sites, which have a high biological and pharmacological importance. COX-1 and COX-2 active sites are constricted by highly conserved residues Arg120/Tyr355 and Ser530/Glu524. In COX-2, the side pocket located above this constriction is delimited by the amino acids Val434, Arg513, and Val523, whereas in COX-1, the side pocket is delimited by different amino acids (Ile434, His514, and Leu523, respectively), indicating spatial changes. Because Val523 is smaller than Leu523, opening the side pocket increases the solvent-accessible surface area at COX-2 active site [29]. The active site of COX-2 is approximately 27% larger than that of COX-1, allowing the synthesis of compounds that specifically interact with the active site of COX-2 without inhibiting the catalytic activity of COX-1 [29]. COX-1 [*prostaglandin-endoperoxide synthase 1* *(PTGS1*) localized in chromosome 9], also classified as constitutive, is expressed in the gastrointestinal mucosa, platelets, endothelium, kidneys, and uterus [34]. In humans, the two isoenzymes feature different properties and are located in different chromosomes [32]. COX-2 (*PTGS2* localized in chromosome 1) is responsible for the maintenance of internal homeostasis and participates in the protection of gastric mucosa, vascular smooth muscle contraction, regulation of glomerular filtration, and platelet aggregation [35]. COX-2 (PTGS2 localized in chromosome 1) is induced by various stimuli, namely, proinflammatory cytokines (IL-1, IL-6, and TNF-α), mitogenic and growth factors, and hormones [14]. Different tissues express different levels of COX-1 and COX-2. COX-2 leads to the production of PGE2, a potent vasodilator, in macrophages, whereas COX-1 leads to the production of thromboxane A2 (TxA2) in platelets, causing vasoconstriction and platelet aggregation.

**Figure-1:**
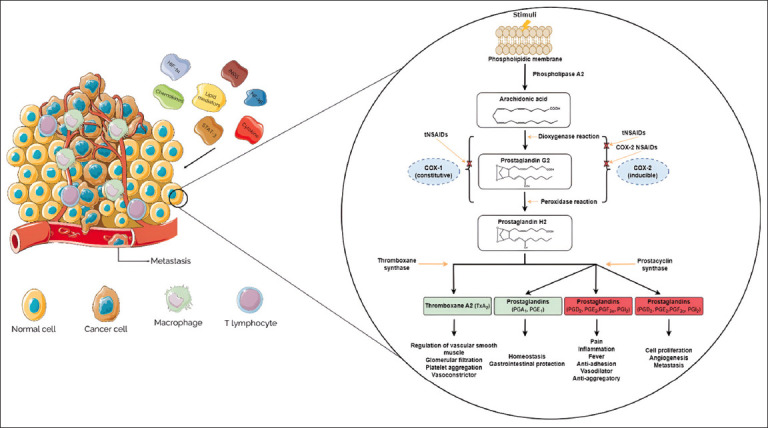
Tumor microenvironment with chronic inflammation and schematic representation of NSAIDs’ mechanism action. COX=Cyclooxygenase enzyme, tNSAIDS=Traditional Non-steroidal anti-inflammatory drugs, COX-2 NSAIDS=COX-2 selective NSAIDs. (Parts of this figure were drawn using pictures from Servier Medical Art, provided by Servier, licensed under a Creative Commons Attribution 3.0 Unported license).

COX-1 and COX-2 catalyze the same reaction for PG production (Figure-1). COX-2 is responsible for producing PGs involved in inflammation, fever, and pain [36]. COX-2 is overexpressed in breast, urinary, colorectal, prostate, and lung cancers [37]. COX-2 exerts a pleiotropic and multifaceted role in the genesis or promotion of carcinogenesis and cancer cell resistance to chemotherapy and radiotherapy. COX-2 is released into the tumor microenvironment by macrophage type 2 cells, cancer-associated fibroblasts, and cancer cells. It induces cancer stem cell-like activity and promotes the proliferation, angiogenesis, inflammation, apoptotic resistance, invasion, and metastasis of cancer cells [38]. Unlike COX-2, there is no evidence that COX-1 is related to the development of chronic inflammation and breast cancer [24]. Moreover, this isoform is also constitutively expressed in the brain, kidney, gastrointestinal tract, thymus, and placenta without being associated with inflammation [39]. COX-2 in the brain plays a role in memory and anxiety, whereas COX-2 influences tissue homeostasis, namely, local renal vasodilation, and improves blood flow in the kidney [39, 40]. Although the constitutive activity of COX-2 is not fully understood, studies with NSAIDs and COX-2 inhibitors have suggested that renal side effects are associated with the inhibition of COX-2 activity in the kidney [41].

The COX-3 enzyme isoform was discovered in the cerebral cortex of the canine brain in 2002 by Daniel Simmons and collaborators. COX-3 is a variant of COX-1 mRNA that retains intron 1, also known as COX-1b or COX-1v. COX-3 has been shown to be selectively inhibited by acetaminophen [42]. COX-3 mRNA has been identified in the hypothalamus, pituitary, and choroid plexus in humans [43, 44]. COX-3 mRNA has been identified in the heart, endothelium, kidney, and neuronal tissues in rodents [43]. No physiological/pathological role has yet been reported in humans or rats [45]. In mice, several studies have hypothesized that COX-3 inhibited by acetaminophen regulates body temperature, producing hypothermia and suggesting antipyretic properties [46, 47]. However, the effects of acetaminophen have been observed in humans, where no functional COX-3 enzyme has been sequenced [45, 48]. The role of COX-3 in dogs is not yet known. Further research is warranted to elucidate functions in hemostasia and pathological conditions, namely, cancer.

### COX-2 and Breast Cancer

Breast cancer is a very complex group of neoplasms arising from epithelial and/or mesenchymal components of the mammary gland tissue [49, 50]. Estimates indicate that approximately 20%–30% of early-stage breast cancer will progress to metastatic disease [51]. Treatment mainly consists of surgical intervention, chemotherapy, radiation, and endocrine management [52]. In addition to humans, mammary tumors develop in other species, such as female dogs and cats [53]. Breast cancer is associated with a high expression of COX-2. Many cancer risk factors, such as nicotine, nitrosamine, radiation, ultraviolet B, free radicals, oncogenic proteins, and growth factors [54], are capable of inducing COX-2 expression. COX-2 overexpression has been associated with increased angiogenesis, tumor invasion, immunosuppression, and decreased apoptosis [9, 38]. In a study with 1576 invasive breast carcinoma samples, COX-2 was detected by immunohistochemistry in 37.4% of samples and was significantly more frequent in ductal carcinomas (39.9%) followed by lobular carcinomas (29.5%). It was correlated with a poor prognosis and unfavorable outcome, including large tumor size, high histological grade, negative hormone receptor status, high proliferation rate, and Human Epidermal Growth Factor Receptor 2 (HER2) oncogene amplification [55]. COX-2 overexpression was also associated with reduced disease-free survival and disease-related survival in estrogen receptor (ER)-negative patients but not in ER-positive patients [56]. A study of 45 breast tumors and 22 normal breast tissue samples concluded that COX-2 mRNA expression was higher in ER-and progesterone receptor (PR)-negative tumors compared with that in hormone-positive tumors [57]. COX-2 overexpression is associated with higher malignancy, recurrence, metastasis, angiogenesis, and lower disease-free and overall survival in canine and feline mammary tumors [58, 59]. COX-2 overexpression was also observed in chemically induced rat mammary tumors [60].

## NSAIDs and Mechanism of Action

**Figure-2:**
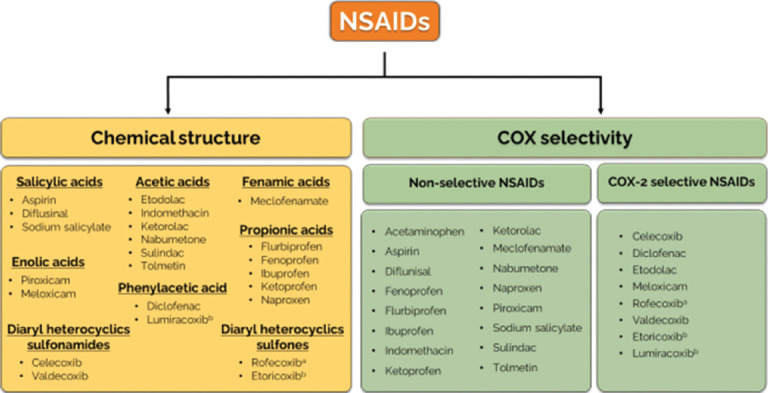
Classification of steroidal anti-inflammatory drugs (NSAIDs), according to their chemical structure and COX isoform selectivity. COX=Cyclooxygenase, NSAIDs=Non-steroidal anti-inflammatory drugs, a. Withdrawn from the market, b. prodrugs

NSAIDs are among the most commonly prescribed drugs worldwide [61, 62]. They constitute a heterogeneous group of drugs with analgesic, antipyretic, antiaggregant, and anti-inflammatory properties and different pharmacokinetic and pharmacodynamic profiles [62]. At present, there are more than 50 different NSAIDs on the global market. NSAIDs are classified as acetic acids, salicylic acids, phenylacetic acids, propionic acids, fenamic acids, diaryl heterocyclic sulfonamides, diaryl heterocyclic sulfones, and enolic acids based on their chemical structure (Figure-2) [63]. NSAIDs can also be classified according to the selective inhibition of COX isozymes as non-selective or traditional NSAIDs, if they inhibit COX-1 and COX-2, or selective, if they only inhibit COX-2 (Figure-2) [63].

NSAIDs are relatively inexpensive and widely available without prescription, which is the main advantage. They are also relatively safe when used as instructed, but like all drugs, side effects can also occur [64]. NSAIDs are associated with gastrointestinal complications, cardiovascular events, and renal failure [41]. The selective inhibition of COX-1 and COX-2 enzymes significantly impacts side effects. In the 1990s, NSAIDs selective for COX-2 were developed in order to avoid the gastrointestinal side effects associated with COX-1 inhibitor. Selective inhibition of COX-2 synthesis and preservation of COX-1 activity are critical factors in reducing adverse gastrointestinal effects caused by NSAIDs [65]. Although COX-2 inhibitors reduce adverse gastrointestinal effects, they are associated with a higher cardiovascular risk (e.g., stroke and heart failure) [66].

NSAIDs have been used for almost two hundred years, but their mechanism of action was only identified in 1971. Arachidonic acid is a polyunsaturated fatty acid released from the phospholipid membrane by phospholipase A2, which can be activated by various stimuli, such as inflammatory, chemical, physical, and mitogenic stimuli [32]. Arachidonic acid may also be released by phospholipidases C or D conjugated with diacylglycerol lipase [67]. Once released, arachidonic acid becomes available to cytochrome P-450 monooxygenase, lipoxygenase, and the COX pathway [68]. Inflammatory pathways are most widely used to study the effects of NSAIDs in cancer research. COX plays a two-fold role in the biotransformation of arachidonic acid and is involved in both dioxygenase and peroxidase reactions in the presence of oxygen and heme products. The first reaction leads to the synthesis of unstable PG2, which incorporates two oxygen molecules on carbons 9 and 1. Subsequently, the intermediate PG2 is converted into PGH2 in a peroxidase reaction, where it is oxidized [67]. PGH2 is a precursor for various prostanoids, such as PGs, prostacyclins, and Txs, through the action of specific isomerases [69]. Prostanoids are produced in response to a variety of stimuli, bind to multiple receptors, change the normal physiological state, and induce disease development. Prostanoids may act in a paracrine or autocrine manner [70]. PGE2 is an important prostanoid implicated in tumorigenesis and the major metabolic product of COX-2. PGE2 is increased in colorectal, liver, cervical, prostate, and breast cancers and is often associated with a poor prognosis [71–73]. In addition, PGE2 activates the vascular endothelial growth factor (VEGF) pathway that induces angiogenesis and favors cellular proliferation, tumor growth, and metastasis [74].

Inhibition of PG synthase is not the only mechanism of action of NSAIDs, which can act on multiple molecular targets. In other words, specific NSAIDs can model other specific signaling pathways, such as NF-κB, 5-lipoxygenase, NSAID-activated gene-1, peroxisome proliferator-activated receptor subtypes α, γ, and δ, the Wnt/β-catenin pathway, cytochrome c, Akt pathway, and mechanistic target of rapamycin [12]. Because these pathways have been identified in breast tumors, it is easy to understand the importance of COX-2 inhibition for cancer control [75–79].

## NSAIDs for Breast Cancer

**Figure-3:**
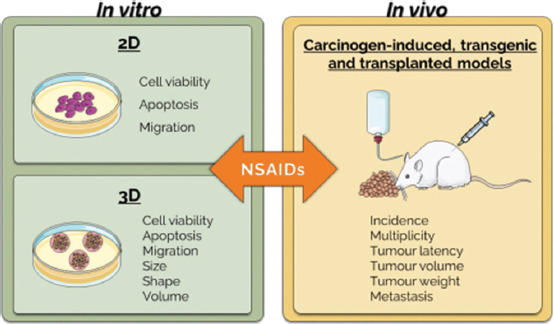
An overview of the key parameters assessed in each breast cancer model (Parts of the figure were drawn by using pictures from Servier Medical Art, provided by Servier, licensed under a Creative Commons Attribution 3.0 Unported license).

Combination therapy with NSAIDs to decrease inflammation can be a therapeutic approach for breast cancer treatment [14]. The relationship between breast cancer and NSAIDs administration has been studied by several researchers, but the results remain controversial (Figure-3) [80, 81].

### *In vitro* studies to assess the efficacy of NSAIDs

In a seminal study, Lasfargues and Ozzello generated the first human breast cancer cell line from a primary invasive ductal breast carcinoma and designated this cancer cell line (BT-20) as basal-like triple-negative breast cancer [82]. At present, all types and subtypes of breast cancer cell lines are available, and their selection is based on the objectives of each experiment [83]. In addition, tumors developed in animals have been used to create cancer cell lines. The first canine mammary cancer cell line (REM 134) was established in 1982 [84], and 3 years later, the first feline mammary cancer cell line (JM) was established from an adenocarcinoma [85]. The first rat mammary cancer cell lines were derived from a 7,12-dimethylbenz[a]anthracene (DMBA)-induced mammary tumor (RAMA 25) [86], whereas the first mouse mammary cancer cell lines were derived from a single mammary tumor in a BALB/cfC3H mouse [87]. However, the number of cell lines obtained from available animal mammary cancer is smaller than that from human breast cancer.

The two-dimensional (2D) model, in which adherent cells grow in a monolayer attached to a plastic surface [88, 89], is the most commonly used type of *in vitro* cell culture. This method has been widely used in cell biology research for several decades and is a fundamental tool for studying the behavior of individual cell types when exposed to different conditions, such as drugs, growth factors, and/or genetic modification [90]. The use of 2D cell cultures allows many assays in a short period of time, enables control of environmental conditions, and is a low-cost method compared with *in vivo* studies [90, 91]. Despite these advantages, two-dimensional models have several limitations because they cannot replicate certain *in vivo* conditions [92]. The main disadvantages include the loss of phenotypic characteristics of the original cells, loss of homology of the biological system outside the natural environment, absence of nerve and endocrine signals, and inability to simulate cell-cell contacts [90]. More recently, three-dimensional (3D) techniques have been developed to overcome these disadvantages. 3D cell cultures have been gaining popularity as a viable approach to bridge the gap between conventional flat cell cultures and animal models [88, 93]. Tissue explants, spheroids, and organoids are some 3D models that have been developed. Spheroids and organoids can also be established with or without support (as extracellular matrix) and designated as scaffold-based and scaffold-free, respectively [93]. *In* *vivo*, architecture, cellular heterogeneity and interactions, and microenvironmental tumor variables can be simulated using 3D culture techniques [94]. 3D cell culture systems exhibit a high level of organization, are characterized by consistent morphology and shape, and preserve cell-cell and cell-matrix interactions [95]. 3D models can be employed to assess cell availability, apoptosis, migration, and morphometric parameters generally determined by tumor mass (e.g., size, shape, diameter, circularity, volume, and cell density).

Breast cancer cell lines have been used in numerous *in vitro* studies to evaluate the effect of NSAIDs on carcinogenesis. Despite all available breast cancer cell lines that represent the entire spectrum of breast tumors identified in women, most studies use Michigan Cancer Foundation-7 (MCF-7) (luminal A subtype) and/or M.D. Anderson-Metastatic Breast-231 (MDA-MB-231) (triple-negative subtype). Because research in veterinary medicine is increasing and many animals are used as human models, *in vitro* studies on human breast cancer cell lines or canine, feline, and murine breast cancer cell lines were included in this review. Celecoxib is one of the most investigated NSAIDs in human-derived breast cancer cell lines [96–101]. Few experimental assays have been performed to evaluate the efficacy of NSAIDs in canine and feline mammary cancer [102–106]. Acetylsalicylic acid, celecoxib, meloxicam, piroxicam, and deracoxib were the most frequently tested NSAIDs in canine mammary cancer cell lines [103–106], whereas acetylsalicylic acid was the only NSAID evaluated in a feline and a murine mammary cancer cell line [102, 107]. NSAIDs, along with natural compounds such as curcumin, luteolin, resveratrol, and phosphatidylcholine [96, 108–110], have been tested in isolation. More recently, NSAIDs modified with metal ions, such as copper [111], silver [111–113], and zinc [114], with organoantimony [115], and conjugated with other antiproliferative agents, such as mitochondriotropic agents (triphenylarsine and triphenylphosphine) [111, 112] have also been investigated. Tables-1 and 2 [96–125] list the *in vitro* studies performed on human breast, canine, feline, and murine mammary cancer cell lines addressing the effects of NSAIDs. *In vitro* studies using 3D human breast cancer models to evaluate the effects of NSAIDs are scarce [116, 117]. No 3D animal mammary cancer models in which NSAIDs were tested have been published in the literature.

**Table-1:** *In vitro* studies performed in human breast cancer cell lines to assess the effects of NSAIDs.

NSAIDs	Cell line	Dose	Effects	Reference
**2D Models**				
Acetylsalicylic acid	MDA-MB-231	0.5–16 mM	↓ Cell viability	[116]
Acetylsalicylic acid	MDA-MB-231-TmxR (tamoxifen resistant)	0.5–16 mM	↓ Cell viability	[116]
Acetylsalicylic acid (with phosphatidylcholine)	MCF-7	0–180 µg/mL	Ø Cell proliferation	[109]
Acetylsalicylic acid (with phosphatidylcholine)	MDA-MB-231	0–180 µg/mL	Ø Cell proliferation	[109]
Acetylsalicylic acid (with phosphatidylcholine)	SK-BR-3	0–180 µg/mL	Ø Cell proliferation	[109]
Acetylsalicylic acid (with silver and Ph_3_Sb) {Ag (Ph_3_Sb)_3_(Asp)}	MCF-7	5.88 μΜ	↑ Cytotoxicity	[113]
Acetylsalicylic acid (with silver and Ph_3_Sb) {Ag (Ph_3_Sb)_3_(Asp)}	MDA-MB-231	14.86 μΜ	↑ Cytotoxicity	[113]
Acetylsalicylic acid (with silver and tpAs) [Ag (Asp)(tpAs)_3_]	MCF-7	5.6 μΜ	↑ Genotoxicity Cell cycle arrest (G1 phases) ↓ LOX activity	[112]
Acetylsalicylic acid (with silver and tpAs) [Ag (Asp)(tpAs)_3_]	MDA-MB-231	3.2 μΜ	↑ Genotoxicity Cell cycle arrest (G1 phases) ↓ LOX activity	[112]
Celecoxib	MCF-7	10, 20, 40 μmol/L	Ø Cell proliferation ↓ COX-2 expression ↓ PGE2 level	[97]
Celecoxib	MCF-7	1, 10, 25, 50, 75 and 100 μmol/L	Ø Cell proliferation	[96]
Celecoxib	MCF-7	20, 40, 60 80 and 100 μM	↓ Cell proliferation ↓ SOX-2 protein level	[98]
Celecoxib	MCF-7	95.44, 49.50 and 97.70 μM	Ø Cell proliferation and migration ↑ Cell apoptosis	[99]
Celecoxib	MDA-MB-231	12.5–50 mM	Ø Cell proliferation	[100]
Celecoxib	MDA-MB-231	20–60 μM	Ø Cell proliferation Cell cycle arrest (G0/G1 phases) ↓ AKT activity ↑ BAX activity ↓ VEGF activity	[101]
Celecoxib	MDA-MB-231	20, 40, 60 80 and 100 μM	↓ Cell proliferation ↓ SOX-2 protein level ↓ *SNAIL*, *SLUG* and *TWIST* expression ↑ E-cadherin protein level ↓ Vimentin protein level	[98]
Celecoxib	MDA-MB-231	95.44, 49.50 and 97.70 μM	Ø Cell proliferation and migration↑Cell apoptosis	[99]
Celecoxib	MDA-MB-231	10, 20, 40 μmol/L	Ø Cell proliferation (dose and time-dependent manner) ↓ COX-2 expression ↓ PGE2 level	[97]
Celecoxib	MDA-MB-468	20–60 μM	Ø Cell proliferation Cell cycle arrest (G0/G1 phases) ↓ COX-2 activity	[101]
Celecoxib	T47D	95.44, 49.50 and 97.70 μM	Ø Cell proliferation and migration ↑ Cell apoptosis	[99]
Celecoxib (alone or combinated with resveratrol)	MCF-7	1–100 µmol/L	↓ Cell viability (dose-dependent and time-dependent manner)	[96]
Celecoxib (with curcumin)	MDA-MB-231	10, 15, 20 and 25 µM	Ø Cell proliferation (dose-dependent manner)	[108]
Celecoxib (with luteolin)	MCF-7	0, 10, 25, 50, 75, 100 µM	Ø Cell proliferation (dose and time-dependent manner) ↑ Cell apoptosis ↓ Akt level	[110]
Celecoxib (with luteolin)	MDA-MB-231	0, 10, 25, 50, 75, 100 µM	Ø Cell proliferation (dose and time-dependent manner) ↑ Cell apoptosis ↓ Akt level	[110]
Celecoxib and nitro-oxy derivative of celecoxib	MCF-7	25 µM and 50 µM	Ø Cell growth ↓ ERα expression	[120]
Diclofenac	MCF-7	3.125, 6.25, 12.5, 25, 50, 100, 200 and 400 µg/mL	Ø Cell proliferation ↑ Cell apoptosis (dose-dependent manner)	[118]
Diclofenac	MDA-MB-231	0.2, 0.4, 0.8 mM	Ø Cell proliferation, ↓ GLUT1 and c-Myc expression ↓ HK activity	[121]
Ibuprofen (loading in micelles composed of amphilic chitosan)	MCF-7	0.101 and 0.065 mg/mL	↓ Cell viability	[122]
Indomethacin (associated phosphatidylcholine)	MCF-7	0-50 µM	Ø Cell proliferation	[109]
Indomethacin (associated phosphatidylcholine)	MDA-MB-231	0-50 µM	Ø Cell proliferation	[109]
Indomethacin (associated phosphatidylcholine)	SK-BR-3	0-50 µM	Ø Cell proliferation	[109]
Mefenamic acid (with zinc and 1,10-phenanthroline -5,6-dione) [(phendione) Zn^II^ (MFN)_2_]	MDA-MB-231	0.7 μM	↓ Cell viability ↑ COX-1 and 2 inhibitions ↓ PGE2 synthesis	[114]
Naproxen derivatives bearing hydrazide-hydrazone: (S)-2- (6-methoxynaphthalen- 2-yl)-N’- {(E)- [2-(tri-fluoromethoxy) phenyl] methylidene} propanehydrazide	MDA-MB-231	22.42 µM (IC_50_)	Ø Cell proliferation; ↓ VEGFR-2 and Bcl-2 expression; Cell cycle arrest (S and M phases)	[119]
Naproxen (with silver and tpAs) [Ag (Nap)(tpAs)_3_]	MCF-7	3.5 μΜ	↑ Genotoxicity Cell cycle arrest (G1 phases) ↓ LOX activity	[112]
Naproxen (with silver and tpAs) [Ag (Nap)(tpAs)_3_]	MDA-MB-231	4.8 μΜ	↑ Genotoxicity Cell cycle arrest (G1 phases) ↓ LOX activity	[112]
Naproxen (with zinc and 1,10-phenanthroline- 5,6-dione) [(phendione) Zn^II^ (NPR)_2_(H_2_O)_2_]	MDA-MB-231	1.5 μM	↓ Cell viability ↓ Cell migration ↓ Vimentin and beta-1 integrin ↑ Caspase 3, 8 and 9 ↑ COX-1 and 2 inhibitions ↓ PGE2 synthesis	[114]
Parecoxib (with sufentanil)	BT474	300 µmol/L (with 1 nmol/L of sufentanil)	Ø Cell proliferation Cell cycle arrest (G1 phases); ↓ Cyclin D1, COX-2, MMP-9, VEGFA, N-cadherin, vimentin and snail expression; ↑ E-cadherin expression	[123]
Piroxicam	MCF-7	10, 20, 30, 50, 100 µM	Ø Cell proliferation ↑ ROS and Akt expression	[124]
Piroxicam	MDA-MB-231	10, 20, 30, 50, 100 µM	Ø Cell proliferation	[124]
Salicylic acid (with copper and TTP) [Cu (SalH)(TPP)_3_]	MCF-7	2.4 μΜ	↓ Cell proliferation ↑ Cell apoptosis ↑ DNA fragmentation	[111]
Salicylic acid (with copper and TTP) [Cu (SalH)(TPP)_3_]	MDA-MB-231	11.6 μΜ	↓ Cell proliferation ↑ Cell apoptosis ↑ DNA fragmentation	[111]
Salicylic acid (with Ph_3_Sb in the presence of hydrogen peroxide)	MCF-7	11.9 μΜ	Cell cycle arrest (G0/G1 phases) ↓ Genotoxicity	[115]
Salicylic acid (with Ph_3_Sb in the presence of hydrogen peroxide)	MDA-MB-231	8.01 μΜ	Cell cycle arrest (G0/G1 phases) ↓ Genotoxicity	[115]
Salicylic acid (with silver and Ph_3_Sb) {Ag (Ph_3_Sb) 3(SalH)}	MCF-7	3.19 μΜ	↑ Cytotoxicity	[113]
Salicylic acid (with silver and Ph_3_Sb) {Ag (Ph_3_Sb) 3(SalH)}	MDA-MB-231	7.26 μΜ	↑ Cytotoxicity	[113]
Salicylic acid [with silver and tpAs] [Ag (SalH)(tpAs)_3_]	MCF-7	4.1 μΜ	↑ Genotoxicity Cell cycle arrest (G1 phases) ↓ LOX activity	[112]
Salicylic acid [with silver and tpAs] [Ag (SalH)(tpAs)_3_]	MDA-MB-231	4.9 μΜ	↑ Genotoxicity Cell cycle arrest (G1 phases) ↓ LOX activity	[112]
Salicylic acid (with silver and TTP) [Ag (SalH)(TPP)_3_]	MCF-7	2.7 μΜ	↓ Cell proliferation	[111]
Salicylic acid (with silver and TTP) [Ag (SalH)(TPP)_3_]	MDA-MB-231	3.5 μΜ	↓ Cell proliferation	[111]
**3D Models**				
Acetylsalicylic acid	MDA-MB-231 (spheroid culture)	0.5-16 mM	↓ Tumor spheroid volume ↓ Tumor spheroid viability	[116]
Acetylsalicylic acid (with metformin, and oseltamivir phosphate)	MDA-MB-231 (spheroid culture)	0.5-16 mM	↓ Tumor spheroid volume ↓ Tumor spheroid viability ↑ Apoptotic activity ↑ Sensitivity to tamoxifen therapy.	[116]
Celecoxib	MDA-MB-231 (*Antheraea mylitta* fibroin scaffolds)	934 μM	↓ Area ↓ Cell viability ↓ VEGF expression ↓ IL-8 expression	[117]

↓=Decrease, ↑=Increase, Ø=Inhibition, Asp=Aspirin, BAX=Bcl-2 associated protein X, Bcl-2=B-cell lymphoma-2, COX-2=Cyclooxygenase 2, ERα=Estrogen receptor alpha, GLUT1=Glucose transporter 1, IL-8=Interleukin-8, HK=Hexokinase, LOX=Lipooxygenase, MMP-9=Matrix metalloproteinase-9, Nap=naproxen, Ph_3_Sb=triphenylstibine, SalH2=Salicylic acid, TpAs=triphenylarsine, TTP=Triphenylphosphine, VEGFA=Vascular endothelial growth factor A, VEGFR-2=Vascular endothelial growth factor receptor 2, NSAIDs=Non-steroidal anti-inflammatory drugs, PG=Prostaglandins

**Table-2:** *In vitro* studies performed in canine and feline mammary cancer cell lines to assess the efficacy of NSAIDs.

NSAIDs	Cell line	Dose	Effects	Reference
**Canine**				
Acetylsalicylic acid	CHMm	2.5, 5, 10 mM	↓ Bcl-2/Bax ratio ↓ Cell viability	[106]
Acetylsalicylic acid	CHMp	2.5, 5, 10 mM	↓ Cell viability	[106]
Celecoxib	AZACB	10–100 mM	Ø Cell proliferation ↑ G2/M arrest ↓ COX-2 expression	[104]
Firocoxib	UNESP-CM5	1–1000 mM	↓ Cell viability ↑ Apoptotic index	[125]
Firocoxib	UNESP-MM1	1–1000 mM	↓ Cell viability ↑ Apoptotic index	[125]
Meloxicam	CF41.Mg	0.25 µg/mL	↓ Cell migration ↓ Invasion ↓ Matrix metalloproteinase-2 and β-catenin expression	[103]
Piroxicam and deracoxib (both single and combined)	CMT-U27	50, 100, 250, 500, and 1000 µM	↓ Cell viability ↑ Apoptotic cell↑G2/M arrest	[105]
**Feline**				
Acetylsalicylic acid	FMCm	50 µM	No inhibitory effects were observed	[102]
**Mice**				
Celecoxib	BJMC3879	20 µM	↑ G1 arrest ↓ S and G2/M phases ↑ Apoptotic index ↑ caspase-3 and -9 activity ↓ Mitochondrial membrane potential ↓ VEGF-A and COX-2 expression ↓ PGE2 level	[107]

BCl-2=B-cell lymphoma, COX-2=Cyclooxygenase, VEGFA=Vascular endothelial growth factor A, PG=Prostaglandins, Ø=Inhibition

We evaluated the effects of celecoxib in MCF-7 and MDA-MB-231 cells at different concentrations (10, 20, and 40 μmol/L) and exposed the cells for 24, 48, 72, and 96 h. We observed the inhibition of breast cancer cell line proliferation in a dose- and time-dependent manner using the 3-(4,5-dimethylthiazol-2-yl)-2,5-diphenyltrazolium bromide assay. Furthermore, celecoxib decreased COX-2 expression and PGE2 levels, arrested the cell cycle in G0/G1, and decreased the number of cells in the S phase [97]. An *in vitro* study was conducted to evaluate the effect of diclofenac on the MCF-7 cell line. Diclofenac at all concentrations (3.125, 6.25, 12.5, 25, 50, 100, 200, and 400 µg/mL) decreased cell proliferation in a dose-dependent manner. Inhibition was stronger after 48 h of exposure than after 24 h. In addition to its antiproliferative properties, cytomorphological analyses (4′,6′-diamino-2-phenylindole stain and acridine orange/ethidium bromide) have demonstrated proapoptotic effects [118]. The effects of acetylsalicylic acid on a canine mammary cancer cell line were also evaluated. Two canine mammary cancer cell lines, CHMp and CHMm, isolated from primary and metastatic lesions, respectively, were used for this purpose. Cells were exposed to 2.5, 5, and 10 mM acetylsalicylic acid for 12, 24, 36, and 48 h. Cell viability is decreased in both CHMp and CHMm cell lines. However, B-cell lymphoma-2 (Bcl-2)/Bcl-2 associated protein X (BAX) ratio decreased only in CHMm [106].

In 2020, Sambi *et al*. evaluated the effects of acetylsalicylic acid in 3D spheroid cultures from MDA-MB-231 cells in isolation and in combination with metformin and oseltamivir phosphate. A reduction in tumor spheroid volume and viability was observed at acetylsalicylic acid doses of 8, 10, and 16 mM for 72 h. The spheroidal volume was also significantly reduced at 4 mM of acetylsalicylic acid. A decrease in both cell viability and tumor spheroid volume was observed with the administration of acetylsalicylic acid at doses of 8, 10, and 16 mM, plus constant oseltamivir phosphate (300 μg/mL) and metformin (4 mM) [116].

The antitumor effects of NSAIDs in *in vitro* models are dose- and time-dependent, as shown in Table-1. The most frequently observed phenomenon is decreased cell viability and/or proliferation [106, 109]. Some studies have analyzed COX-2 and PGE2 to determine whether the anticancer effect is due to a specific disruption pathway [97, 101, 114]. The Lipooxygenase pathway has also been investigated to examine whether cell death can be attributed to this specific pathway [112]. In addition, apoptosis studies incorporate evaluations of BAX and Bcl-2 proteins [101, 106, 119].

### *In vivo* studies to assess the efficacy of NSAIDs

Animal models enable the study of carcinogenesis pathways and/or the performance of pre-clinical studies to evaluate the efficacy of different chemopreventive and/or therapeutic compounds [126]. Therefore, rodent models of mammary carcinogenesis may be obtained through the administration of chemical carcinogens to increase the incidence rates of mammary tumors and accelerate mammary cancer development [127]. Transgenic or transplanted models are another option to study breast cancer in murine models [128]. Induction of carcinogenesis, mainly chemical induction, is the most commonly used method for the experimental study of mammary cancer [127, 129]. *N*-methyl-*N*-nitrosourea (MNU) and DMBA are the most frequently used carcinogens, enabling the development of mammary neoplasms a few weeks after a single injection [129]. MNU is an alkylating agent that methylates guanine nucleosides and promotes mutations. DMBA is a polycyclic aromatic hydrocarbon that forms an adduct in DNA after cytochrome P450 bioactivation [129]. Published studies indicate that MNU generates more aggressive mammary tumors than DMBA in a shorter time [130]. Sprague-Dawley and Wistar rats are the most commonly used strains in mammary carcinogenesis because they are more susceptible to carcinogenic agents than other strains [131]. A broad spectrum of hormone-positive histological lesions has been observed in rat models of chemically induced mammary tumors [132]. Similar to *in vitro* assays, celecoxib is the most evaluated NSAID in *in vivo* models of mammary cancer. In this study, we evaluated the *in vivo* effects of celecoxib and its combination with resveratrol in female Sprague–Dawley rats at 50 mg/kg on their 43^rd^ and 50^th^ postnatal days. MNU chemoprevention with resveratrol, celecoxib, or a combination of both was initiated 2 weeks before the first carcinogen administration and was administered for 16 weeks. Celecoxib was added to pellets containing 1.67 g/kg of food (0.167%) and administered *ad libitum*. Resveratrol (100 mg/kg) was dissolved in 10% ethanol and administered gavage. Celecoxib alone significantly prolonged tumor latency and decreased the total number of tumors compared with the control group. In addition, the combination of resveratrol and celecoxib reduced tumor frequency by 29% compared with celecoxib alone [96]. The therapeutic effects of ibuprofen and celecoxib in female Sprague–Dawley rats after DMBA administration were also evaluated in another study. A standard diet supplemented with 1500 mg/kg celecoxib (1500 ppm) and ibuprofen (1500 mg/kg) was given to one group of animals. Both celecoxib and ibuprofen reduced the incidence, frequency, and volume of mammary tumors; however, celecoxib was the most effective treatment [133].

The term “genetically engineered models” corresponds to animal strains with genetic modifications that can be categorized as transgenic, knock-in, or knock-out, depending on whether DNA sequences have been added, modified, or removed [134]. The mouse mammary tumor virus/c-Myc model was the first transgenic breast cancer mouse model reported in 1984, in which overexpression of the Myc transcription factor in the mammary gland resulted in spontaneous mammary adenocarcinomas [135]. However, MTV/*neu* and HER2/*neu* have been used in the literature to evaluate NSAIDs [136, 137]. Celecoxib was again tested. Lanza-Jacoby *et al*. used female HER2/*neu* homozygotes (expressing non-transforming rat proto-oncogene) mice to evaluate the effects of celecoxib in mammary tumors. Four-week-old mice fed a diet supplemented with 900 ppm celecoxib. Animals were sacrificed when the tumors reached 20 mm in diameter or 15 months old. Celecoxib decreased tumor incidence and multiplicity, prolonged tumor latency, reduced lung metastasis, and PGI2 and PGE2 concentrations in mammary tumors and their adjacent mammary glands [137].

Transplanted animal models were obtained by transplanting a cancer cell line or solid tumors from a donor. According to transplant source, these models can be divided into syngeneic or xenograft models [138, 139]. Syngeneic approaches use cells and hosts from the same inbred genetic background and do not require immunocompromised hosts. Xenograft approaches use immunosuppressed animals because tumor donors (e.g., humans, dogs, and cats) and hosts (e.g., mice or rats) are from different species [140]. Breast/mammary cancer cells or tissues can be transplanted at the original site (orthotopic) or alternative sites (heterotopic) [134]. In 1962, the first xenograft breast cancer model was reported by heterotransplantation of a human breast cancer cell line into an immunodeficient mouse [141]. In transplant models, various animal strains have been used, with acetylsalicylic acid, celecoxib, deracoxib, diclofenac, indomethacin, and piroxicam being tested. The effects of celecoxib and diclofenac were evaluated using a syngeneic model. The authors subcutaneously injected 1 × 10^6^ mouse mammary tumor cells (Ehrlich carcinoma cells) into adult female Swiss mice. Twelve days after cell implantation, celecoxib (25 mg/kg) and diclofenac (12.5 mg/kg) alone or in combination with doxorubicin (2 mg/kg) were observed. After 10 days of treatment, the animals were sacrificed and tumor growth delay and volume, changes in tumor DNA content and nitric oxide levels, immunohistochemical staining for p53, and apoptotic index were evaluated. Celecoxib and diclofenac alone showed no significant difference compared with the control group. However, when celecoxib and diclofenac were injected together with doxorubicin, a significant decrease in tumor volume, DNA content and a significant increase in nitric oxide levels and apoptotic index were observed [142]. Yang *et al*. (2017) evaluated the effects of acetylsalicylic acid in canine mammary tumor cells (CHMm) in a xenograft model. Female Balb/c-nude mice were used, and CHMm cells were subcutaneously injected into the right-sided axilla of each mouse. One week after implantation, acetylsalicylic acid (100 mg/day) was i.p. administered to the animals for 2 weeks. The treated group demonstrated a significant decrease in tumor volume and weight compared to the control group. However, acetylsalicylic acid had no effect on diet intake or animal weight. A higher level of fibrosis was also observed in the tumor sections in the treated group [106].

The *in vivo* studies described in this review are restricted to rodent models of mammary cancer in which NSAIDs are administered as chemopreventive and/or therapeutic agents. Table-3 [96–98, 101, 107, 123, 133, 136, 137, 142–146, 147–157] summarizes the main *in vivo* studies conducted and the observed results. Reducing the incidence, multiplicity, and volume of tumors has been the primary focus [133, 143–145]. However, the molecular mechanisms of COX-2 and PGE2 pathways have been poorly explored [96, 136, 146]. Moreover, in addition, the antiangiogenic effects of NSAIDs have also been assessed by VEGF [107].

**Table-3:** *In vivo* studies to assess the efficacy of NSAIDs.

Drug	Model		Dose	Effects	References
**Chemical induction model**					
Celecoxib	♀ Sprague- Dawley Rats	Chemically- induced by DMBA	1500 ppm p.o. in diet for 6W	↓ Tumors volume	[147]
Celecoxib	♀ Sprague- Dawley Rats	Chemically- induced by DMBA	1500 mg/kg celecoxib (1500 ppm) p.o. in diet for 15W	↓ Tumor incidence, multiplicity and volume	[133]
Celecoxib	♀ Sprague- Dawley Rats	Chemically- induced by DMBA	200, 500, 1000 and 1500 ppm p.o. in diet for 122 day (starting 10 days before carcinogen administration)	↓ Tumor incidence at higher dose	[148]
Celecoxib	♀ Sprague- Dawley Rats	Chemically- induced by DMBA	500 mg/kg (500 ppm) and 1500 mg/kg (1500 ppm) o.p. in diet	↓ Tumor incidence, multiplicity and growth	[143]
Celecoxib	♀ Sprague- Dawley Rats	Chemically- induced by DMBA	1000 mg/kg in the oleum maydis	↓ Tumor incidence	[97]
Celecoxib	♀ Wistar rats	Chemically- induced by DMBA	20 mg/kg in combination with 0.5 mL fish oil; p.o. for 7 days followed by DMBA	↓ NF-kB expression ↓ COX-2 level ↓ Cytokines levels	[146]
Celecoxib	♀ Sprague- Dawley Rats	Chemically- induced by MNU	1500 ppm p.o. in diet for 23W	↓ Tumor incidence, multiplicity and body weight	[144]
Celecoxib	♀ Sprague- Dawley Rats	Chemically -induced by MNU	1.666 g/kg diet for 20W (starting a week before carcinogen administration)	↓ Tumor incidence and frequency.	[145]
Celecoxib	♀ Sprague- Dawley Rats	Chemically -induced by MNU	1.67 g/kg (0.167%) p.o. in diet for 16W (2W before the first MNU administration)	↓ Tumor frequency, prolonged tumor latency, and tumor multiplicity ↓ COX-2 expression ↑ GDF15 protein	[96]
Ibuprofen	♀ Sprague- Dawley Rats	Chemically -induced by DMBA	1000 mg/kg rodent diet for 5W	↓ Tumor volume	[149]
Ibuprofen	♀ Sprague- Dawley Rats	Chemically -induced by DMBA	1000 mg/kg rodent diet for 35 days	↓ Tumor volume and inhibited gene expression of both COX-1 and COX-2	[150]
Ibuprofen	♀ Sprague -Dawley Rats	Chemically -induced by DMBA	500 mg/kg ibuprofen (1500 ppm) p.o. in diet for 15W	↓ Tumor incidence, multiplicity and volume	[133]
Naproxen	♀ Sprague -Dawley Rats	Chemically -induced by MNU	400 ppm p.o. in diet, 4 days after MNU injection and for 50W	No inhibitory effects were observed	[151]
Piroxicam	♀ Sprague -Dawley Rats	Chemically -induced by DMBA	0.01% piroxicam in a high-fat (20% soybean oil) or low-fat (0.5% soybean oil) diet	No inhibitory effects were observed	[152]
Rofecoxib	♀ Sprague -Dawley Rats	Chemically -induced by MNU	0.01 mg/1 g (0.001%) and 0.05 mg/1 g (0.005%) in diet 4 days after MNU injection and for 17W	↓ Tumor incidence and tumor volume in both concentrations	[153]
**Transgenic model**					
Celecoxib	♀ MTV/*neu*	Transgenic	500 ppm p.o. in diet for 50W	↓ Tumor incidence and prostaglandin E2 levels	[136]
Celecoxib	HER2/*neu* mice	Transgenic	900 ppm p.o. in diet for 14 months or tumor reached 20 mm diameter	↓ Tumor incidence, multiplicity, prolonged tumor latency and lung metastasis	[137]
**Syngeneic model**					
Celecoxib	♀ Balb/cfC3H	Syngeneic orthotopic: Cell line 410	5 mg/kg/day by gavage for 14 days	↓ Tumor incidence	[154]
Celecoxib	♀ Balb/cfC3H	Syngeneic orthotopic: Cell line 410.4	5 mg/kg/day by gavage for 14 days	↓ Tumor volume ↓ Lung metastasis	[154]
Celecoxib	C3H7He mice	Syngeneic heterotopic: adiministration s.c. of MCa-35	50 mg/kg - 5 times of day intragastric administration	Ø Tumor growth and reveled an antiangiogenic activity	[155]
Celecoxib (with doxorubicin)	♀ Swiss albino mice	Syngeneic heterotopic: s.c. with Ehrlich carcinoma cells	25 mg/kg of celecoxib+2 mg/kg of doxorubicin A single dose 12 days after tumor implantation cells	↓ Tumor volume ↓ DNA content ↑ Apoptotic index ↑ Nitric oxide levels in tumor tissue	[142]
Celecoxib	♀ Fischer rats	Syngeneic orthotopic: MATB	75 mg/kg (1500 ppm) p.o. in diet for 28 days	↓ Metastases	[156]
Celecoxib	♀ BALB/c mice	Syngeneic heterotopic: s.c. with BJMC3879 cells	7.5 and 15 mg/kg i.p. five times per week for 7W.	↓ Tumor volume ↓ Lung and lymph nodes metastases ↓ DNA synthesis ↑ Apoptotic index ↓ Microvessel density ↓ VEGF-A and COX-2 expression	[107]
Diclofenac (with doxorubicin)	♀ Swiss albino mice	Syngeneic heterotopic: adiministration s.c. of Ehrlich carcinoma cells	12.5 mg/kg of diclofenac+2 mg/kg of doxorubicin A single dose 12 days after tumor implantation cells	↓ Tumor volume ↓ DNA content ↑ Apoptotic index ↑ Nitric oxide levels in tumor tissue	[142]
Indomethacin	♀ Balb/cfC3H	Syngeneic orthotopic: Cell line 410	1 mg/kg/day by gavage for 14 days	↓ Tumor incidence	[154]
Indomethacin	♀ Balb/cfC3H	Syngeneic orthotopic: Cell line 410.4	1 mg/kg/day by gavage for 14 days	↓ Tumor volume↓Lung metastasis	[154]
Parecoxib (with sufentanil)	♀FVB/n	Xenografts orthotopic: Tumor of genetically modified mice - FVBMMTV- PyMT	5 mg/kg (with 1 mg/kg of sufentanil)	↓ COX-2, MMP-9, VEGFA, N-cadherin, and snail expression; ↓ Number pulmonary metastasis and tumor growth	[123]
**Xenografts**					
Acetylsalicylic acid	♀ Balb/c-nude	Xenografts orthotopic: CHMm (canine mammary tumor cell line)	25 mg/kg i.p. for 3W (one per day)	↓ Tumor volume	[106]
Celecoxib	♂ Athymic nude mice	Xenograft heterotopic: S.c. with MDA-MB-231 in Matrigel	25 mg/kg (start 7 days before the tumor cells inoculation and during for 52 days; administration method not reported)	↓ Tumor weight ↓ Vascularization ↑ Necrosis in tumor mass	[101]
Celecoxib	♀ NOD/SCID mice	Xenografts orthotopic: MDA-MB-231	30 mg/kg daily by gavage for 30 days	↓ Tumor volume ↓ Tumor weight ↓ β-catenin, p-GSK-3b, MMP-2, Survivin and SOX-2 protein level ↓ *C-myc*, *cyclin -D1* and *axin-2* expression ↓ PGE2 serum level	[98]
Celecoxib	♀ NOD/SCID mice	Xenografts heterotopic: MDA-MB-231 by tail vein injection	30 mg/kg daily by gavage for 20 days	↓ Metastasis lesions on lung and liver	[98]
Deracoxib	♀ nude (unidentified strain)	Xenografts orthotopic: CMT-9 (canine mammary tumor cell line)	0.6 mg/kg daily by gavage for 24 days	No inhibitory effects were observed	[157]
Piroxicam	♀ nude (unidentified strain)	Xenografts orthotopic: CMT-9 (canine mammary tumor cell line)	0.6 and 0.9 mg/kg daily by gavage for 24 days	↓ Tumor volume	[157]

♂=Male, ♀=Female, ↓=Decrease, ↑=Increase, Ø=Inhibition, COX-2=Cyclooxygenase-2, DMBA=7,12-dimethylben anthracene, GFD15=Growth differentiation factor 15, HER2=Human epidermal growth factor-2, MMP=Matrix metalloproteinase, MNU=*N*-methyl-*N*-nitrosourea, i.m.=Intramuscular Injection, i.p.=Intraperitoneal injection, PGE2=Prostaglandin E2, p.o.=Oral administration, s.c.=Subcutaneous injection, VEGFA=Vascular endothelial growth factor A, W=Weeks

### Epidemiological studies

In addition to experimental studies using cancer cell lines or animal models of mammary cancer, many epidemiological studies have examined the effects of these drugs on breast cancer.

A case–control study with 1442 cases and 1420 controls, published in 2004, determined the association between the frequency and duration of acetylsalicylic acid and acetaminophen use and breast cancer risk. This study included women aged 20–98 years who were diagnosed with *in situ* or invasive breast cancer and taking NSAIDs at least once *per* week for 6 months or longer. Acetaminophen is not associated with a lower risk of breast cancer. Acetylsalicylic acid reduces the risk of breast cancer recurrence in women with positive hormone receptor (ER+ and PR+) tumors but not in women with negative hormone receptor-negative tumors. In addition, the inverse association with acetylsalicylic acid was similar between pre- and postmenopausal women [158].

Curiously, Marshal *et al*. concluded that acetylsalicylic acid is inversely associated with hormone-positive tumors but not hormone-negative tumors, only among postmenopausal women. They analyzed 114,460 women aged 22–85 years and free of breast cancer between 1995 and 1996. During the follow-up period between 1995 and 2001, 2391 women were diagnosed with breast cancer. Ibuprofen administration and long-term daily acetylsalicylic acid use were also associated with an increased risk of breast cancer [159]. Another study assessed the association between regular NSAIDs administration and breast cancer risk with a cohort of 7006 breast cancer cases and 3906 healthy controls (1976–2002) aged 30–79 years. Salicylates, indoles, propionic acids, fenamates, pyrazolines, oxidans, and COX-2 inhibitors were used in this study. It was found that the regular use of NSAIDs decreased the risk of breast cancer, which was more pronounced among premenopausal women. In addition, the type of NSAIDs and hormone receptor status did not influence the risk of breast cancer development [160].

Researchers have previously conducted a study where they genotyped 1,067 breast cancer cases and 1,110 control individuals. The aim of this study was to determine whether polymorphisms may reduce overall breast cancer risk or risk of breast cancer subtypes by modulating the inflammatory response and whether they can interact with NSAIDs use. These data prove that NSAIDs can interact to reduce the risk of hormone receptor-positive breast cancer for those with at least one variant C-allele of COX-2. 8473 [161]. However, we did not observe any corresponding interaction with subjects taking only acetylsalicylic acid. This study suggests that genetic polymorphisms in the COX-2 gene could influence the effect of NSAIDs (except acetylsalicylic acid) in breast cancer.

A research team conducted a study in 591 postmenopausal women aged 55–69 years to evaluate the association between self-reported NSAIDs use (acetylsalicylic acid or non-acetylsalicylic acid NSAIDs) and survival after invasive breast cancer diagnosis. These authors observed that NSAIDs reduced breast cancer mortality; however, there was no trend toward decreased deaths by increasing the frequency of NSAID administration [162]. A further study concluded that NSAIDs could reduce the risk of breast cancer development. However, this reduction does not depend on the hormone receptor status [163]. On the other hand, a study aimed to evaluate the effects of acetylsalicylic acid and non-acetylsalicylic acid NSAIDs on ER status concluded that daily acetylsalicylic acid intake was associated with a slight reduction in ER-positive breast cancer. However, this finding did not prove statistically significant. Furthermore, acetylsalicylic acid and non-acetylsalicylic acid NSAIDs are associated with the risk of ER-negative breast cancer [164].

One study published in 2009 observed 112,292 cancer-free women aged 25–42 years. After 14 years, 1345 women have developed invasive premenopausal breast cancer. This study concluded that regular use of acetylsalicylic acid, acetaminophen, or other non-steroidal anti-inflammatory drugs was not associated with a reduced risk of breast cancer development among premenopausal women. Moreover, this study observed that these results were independent of frequency (days *per* week), dose (tablets *per* week), or duration of use [165].

In another study, 1170 breast cancer cases and 2115 controls aged 35–79 years were selected to investigate the association of adult lifetime acetylsalicylic acid, ibuprofen, and acetaminophen intake with breast cancer risk. NSAIDs frequency was categorized into non-user (0 pills/day), low (<2 pills/day), and high (≥2 pills/day) groups. Acetylsalicylic acid administration was inversely associated with breast cancer risk, especially in women aged 61–70 years, with a strong association among those who took ≥2 pills/day. The same association was not observed between ibuprofen and acetaminophen use [166]. The same team also studied the association between acetylsalicylic acid and ibuprofen on the molecular subtypes of breast cancer in the same cohort. Breast cancers were classified according to HER2 protein expression, p53 mutation status, or joint ER, PR, and HER2 status. In the latter case, the breast cancers were subcategorized into the following four groups: luminal subtype was ER-and PR-positive, luminal A-positive for HER2, and luminal B-negative for HER2. HER2-enriched tumors were only HER2-positive and triple-negative for tumors that did not express ER-, PR-, or HER2-related markers. These findings support the hypothesis that acetylsalicylic acid is inversely associated with breast cancer risk. However, this phenomenon was not observed independently of the tumor subtype. Furthermore, acetylsalicylic acid was not associated with HER2 and p53 tumors. Ibuprofen was associated with a significantly increased risk for hormone-positive, HER2, and p53 breast cancers. Ibuprofen has also been shown to be associated with an increased risk of luminal A and B tumors but a decreased risk of HER2-enriched tumors [167].

A study [168] evaluated the effects of acetylsalicylic acid and non-acetylsalicylic acid NSAIDs to determine whether hormone receptor status differs among patients at risk of postmenopausal breast cancer. The authors used a cohort of 41,836 postmenopausal women between 55 and 69 years of age, and after 13 years, 26,580 postmenopausal women were identified as having breast cancer. This study found that acetylsalicylic acid was associated with a reduced risk of postmenopausal breast cancer, independent of ER and PR status. This reduction was more pronounced in patients who took it 6 or more times/week than in those who did not take it [168].

In another study, which investigated women with sisters who had breast cancer, the authors recorded that among 50,884 women who participated in the study, 2118 developed breast cancer. This study included four groups: acetylsalicylic acid, acetyl silicate derivates, coxib NSAIDs, COX-2 inhibitors, non-acetylsalicylic acid, and non-coxibs that did not belong to the acetylsalicylic acid or coxib group. Non-acetylsalicylic acid and acetylsalicylic acid groups were associated with a reduced risk of breast cancer among premenopausal women. However, in postmenopausal women, there was no reduction in the risk of breast cancer with non-acetylsalicylic acid and non-coxib NSAIDs. This study concluded that the use of NSAIDs for chemoprevention may be beneficial in people at a higher risk of breast cancer, such as those who have a sister with the disease [169]. A meta-analysis performed by De Pedro *et al*. suggested a protective effect of NSAIDs, namely, acetylsalicylic acid and COX-2 inhibitors, against breast cancer. However, this study only suggests these findings for hormone-positive tumors. Furthermore, this study did not investigate the effects of different NSAIDs doses or durations [170].

The effects of NSAIDs on three breast cancer subtypes (hormone-positive, HER2+, and triple-negative) were investigated [80]. NSAIDs were divided into the following five groups: Acetylsalicylic acid, acetic acid derivatives, propionic acid derivatives, COX-2 inhibitors, and other NSAIDs. This study involved 1736 breast cancer cases and 1895 healthy controls between 20 and 85 years of age and reported that the administration of acetic acid derivatives, propionic acid derivatives, and COX-2 inhibitors was associated with a 24% reduction in breast cancer development. These results were similar in postmenopausal and premenopausal women. However, there was no risk of reduced cancer development in patients treated with acetylsalicylic acid. In addition, the protective effect was observed only in hormone-positive and HER2+ cancers but not in advanced clinical stages and triple-negative breast cancers [80].

A meta-analysis of observational studies conducted between 1989 and 2019 addressed the effects of acetylsalicylic acid on breast cancer in 2021. Acetylsalicylic acid decreases the risk of breast cancer in hormone receptor-positive, *in situ* tumors, and postmenopausal women. Regular dose (325 mg) and use of acetylsalicylic acid for more than 3 years have also been associated with decreased risk of breast cancer [171].

Many studies [14–16, 160, 161] have shown that NSAIDs have a protective effect against breast cancer; however, the relationship between NSAID use and the risk of developing breast cancer is complex and contradictory. Some studies suggest that hormone status does not have any effect, whereas others indicate the opposite. In short, NSAIDs may be associated with a reduction in risk and mortality in certain subtypes (hormone-positive) and an increase in other subtypes (hormone-negative). In addition, menopausal status is another factor with controversial results. Epidemiological studies need to standardize parameters such as cohorts, NSAIDs classes, doses, frequency, and duration.

## Conclusion

The main goal of this review was to provide the readers with an overview of the potential use of NSAIDs and selective COX-2 inhibitors for breast cancer treatment, highlighting the *in vitro* and *in vivo* studies employed in this field. COX-2 overexpression is observed in several types of cancer, including urinary, colorectal, prostate, lung, and breast cancer, and is associated with a poor prognosis and advanced clinical features. NSAIDs target COX-2, inhibiting the eicosanoid pathway and thus preventing PG synthesis. *In vitro*, *in vivo*, and epidemiological studies have provided cumulative evidence that pharmacological inhibition of COX-2 has a protective effect on breast tumor development. However, epidemiological studies are inconsistent and controversial due to the etiology of different breast cancer subtypes and menopause status. Therefore, the actual role of NSAIDs in the development of breast cancer remains unclear. Further studies with appropriate cohorts and/or matched case–control studies are warranted to unravel the impact of NSAIDs on this disease. However, due to ethical issues, *in vitro* and *in vivo* models are essential tools to understand the interplay between NSAIDs and breast cancer. *In vitro* and syngeneic/xenograft models allow the study of breast cancer subtypes, and chemically induced models should be chosen if hormone-positive tumors are the focus of the study. In line with the “One Health” concept, human and veterinary medicine should share studies for mutual benefit.

Further research is required to determine the effects of NSAIDs on breast cancer, whether isolated or associated with other compounds, as preventive or therapeutic effects. Acetylsalicylic acid and celecoxib were the most evaluated drugs *in* both *in vitro* and *in vivo* studies. Several NSAIDs have not yet been evaluated; therefore, future studies are needed to understand their possible applications in breast cancer research and potential future treatments. *In vitro* 3D models are a promising tool due to their ability to recapitulate tissue architecture, provide physiological relevance, and model disease. Following European and Food and Drug Administration guidelines for the use of animal models, increased *in vitro* research with cocultures (e.g., with cancer-associated fibroblasts) and 3D models to evaluate the efficacy and safety of NSAIDs is a certified alternative. Because NSAIDs act as proliferation and angiogenesis inhibitors, their combined administration with classical anti-neoplastic drugs may help reduce the toxicity of NSAIDs and improve patients’ quality of life. In addition, NSAIDs may be administered in combination with natural anti-inflammatory substances (for example, plant extracts) to reduce side effects associated with long-term use of NSAIDs. Administration of NSAIDs incorporated into nano-delivery systems to improve drug stability, prolong circulation time, and improve targeting to specific tissues or cells should also be explored.

## Authors’ Contributions

TF: Conceptualization, writing-original draft, and preparation of the figures. AIF and VMG: Reviewed and edited the manuscript. PAO, JFM, and RM: Designed the manuscript, supervised, reviewed, and edited the manuscript. All authors have read, reviewed, and approved the final version of the manuscript.
